# Regiospecific Grafting of Chitosan Oligomers Brushes onto Silicon Wafers

**DOI:** 10.3390/ijms23148013

**Published:** 2022-07-20

**Authors:** Cyrielle Garreau, Corinne Gablin, Didier Léonard, Thierry Delair, Guillaume Sudre, Stéphane Trombotto

**Affiliations:** 1 Univ Lyon, CNRS, UMR 5223, Ingénierie des Matériaux Polymères, Université Claude Bernard Lyon 1, INSA Lyon, Université Jean Monnet, F-69622 Villeurbanne, France; garreau.cyrielle@gmail.com (C.G.); thierry.delair@univ-lyon1.fr (T.D.); guillaume.sudre@univ-lyon1.fr (G.S.); 2 Univ Lyon, CNRS, Université Claude Bernard Lyon 1, Institut des Sciences Analytiques, UMR 5280, 5, Rue de la Doua, F-69100 Villeurbanne, France; corinne.gablin@isa-lyon.fr (C.G.); didier.leonard@univ-lyon1.fr (D.L.)

**Keywords:** chitooligosaccharide, nitrous acid, depolymerization, functionalization, surface

## Abstract

The functionalization of surfaces using chitosan oligomers is of great interest for a wide range of applications in biomaterial and biomedical fields, as chitosan oligomers can provide various functional properties including biocompatibility, wetting, adhesion, and antibacterial activity. In this study, an innovative process for the regiospecific chemical grafting of reducing-end-modified chitosan oligomers brushes onto silicon wafers is described. Chitosan oligomers (COS) with well-defined structural parameters (average DP ~19 and DA ~0%) and bearing a 2,5-anhydro-d-mannofuranose (amf) unit at the reducing end were obtained via nitrous acid depolymerization of chitosan. After a silanization step where silicon wafers were modified with aromatic amine derivatives, grafting conditions were studied to optimize the reductive amination between aldehydes of amf-terminated COS and aromatic amines of silicon wafers. Functionalized surfaces were fully characterized by AFM, ATR-FTIR, ellipsometry, contact angle measurement, and ToF-SIMS techniques. Smooth surfaces were obtained with a COS layer about 3 nm thick and contact angle values between 72° and 76°. Furthermore, it was shown that the addition of the reducing agent NaBH_3_CN could positively improve the COS grafting density and/or led to a better stability of the covalent grafting to hydrolysis. Finally, this study also showed that this grafting process is also efficient for chitosan oligomers of higher DA (i.e., ~21%).

## 1. Introduction

Chitosan, a natural polysaccharide extracted from biomass, is a copolymer comprising d-glucosamine (GlcN) and *N*-acetyl-d-glucosamine (GlcNAc) units linked by β-(1→4) glycosidic bonds [1]. Industrially obtained by *N*-deacetylation of chitin, chitosan is characterized by its degree of *N*-acetylation (DA) corresponding to the molar ratio of acetylated moieties in the macromolecule. Chitosan can be processed in various physical forms, such as nanoparticles [1], hydrogels [2], and films [3], which demonstrates its versatility. Well-known for its biodegradability, biocompatibility and bioactivity, chitosan has been studied in numerous applications including food, cosmetics, agriculture, or biomedical fields [4,5,6,7,8]. However, its poor solubility in neutral and alkaline pH solutions and its high viscosity in acidic aqueous solutions may impede the use of chitosan in biomaterial coating applications. To overcome this problem, chitosan can be depolymerized by formally hydrolyzing the glycosidic bonds to obtain products with lower degrees of polymerization (DP). When the DP is lower than about 20, the products are usually referred to as chitosan oligomers, chitooligomers, or chitooligosaccharides (COS). There is an increasing interest in COS due to their low molar mass, their better water solubility, and their low viscosity [9]. Chitosan oligomers are also reported to exhibit versatile biological activities similar to chitosan such as antibacterial, antitumoral, cholesterol-lowering, and immune-enhancing effects [10,11,12,13,14]. They are then more suitable for some industrial applications such as food or biomedicine. However, physicochemical and biological properties of COS are strongly influenced by their DP and DA, their pattern of *N*-acetylation, and thus by the preparation methods [15]. Indeed, chitosan oligomers can be produced by enzymatic, physical, or chemical methods. Physical pathways which consist in irradiation with low-frequency ultrasound are rarely employed because of partial depolymerization [10,12,13]. Enzymatic methods consist in hydrolyzing chitosan thanks to enzymes such as cellulases, lipases, proteases, chitinases, or chitosanases [5,10,12,13,15,16]. Finally, chemical methods for the depolymerization of chitosan mainly use hydrochloric acid hydrolysis, nitrous acid deamination, fluorolysis in anhydrous hydrogen fluoride, or oxidative–reductive reactions [5,10,12,13,15,16].

When grafted on surfaces, chitosan brushes in polymer or oligomer form can provide great antibacterial properties [17]. Moreover, as weak polyelectrolytes, chitosan brushes are sensitive to external stimuli such as pH, ionic strength, or counterion size and type [18]. Chitosan brushes can thus have tunable swelling properties that can be useful for controlling drug release and modulating cell adhesion. However, the poor stability of polyelectrolyte coatings via electrostatic interactions limited their use for some applications. In this context, chemical grafting of chitosan brushes onto surfaces is probably the most appropriate strategy to increase the stability of interactions between chitosan and surfaces. A well-known method for the chemical grafting of chitosan is based on the reactivity of the amine groups of the GlcN repeating unit [17,18,19,20,21,22]. However, physicochemical and biological properties of chitosan are closely related to the amount of free amine groups in the macromolecular chain. For this reason, chemically grafting chitosan onto surfaces via the aldehyde group available at their reducing end seems a promising strategy to keep amine groups available and consequently to preserve chitosan physicochemical and biological properties. However, to the best of our knowledge, the regiospecific grafting of chitosan brushes onto surfaces via their reducing ends has not been reported in the literature yet, despite previous studies mentioning chitosan-based advanced materials using reducing-end modified chitosan oligomers [23,24,25,26,27]. Therefore, the main objective of this work is to develop a novel and simple method for the regiospecific functionalization of silicon surfaces using end-modified chitosan oligomers brushes, not depending on the DA or the DP.

**Scheme 1:**
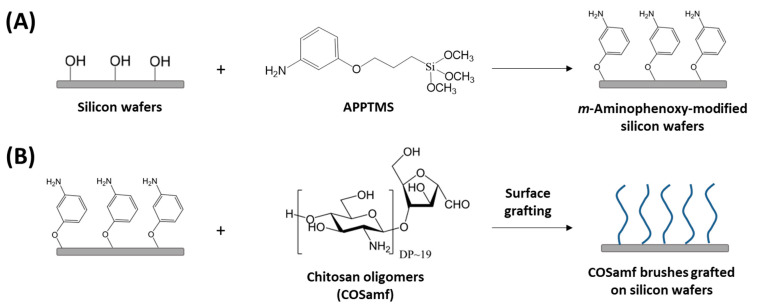
(**A**) Silanization of silicon wafers with 3-(*m*-aminophenoxy)propyltrimethoxysilane (APPTMS) and (**B**) grafting of fully *N*-deacetylated amf-terminated COS (COSamf) brushes to *m*-aminophenoxy-modified silicon wafers via their reducing-end aldehydes.

In this study, fully *N*-deacetylated COS was prepared by nitrous acid deamination of chitosan and grafted onto *m*-aminophenoxy-modified silicon wafers via their 2,5-anhydro-d-mannofuranose (amf) reducing-end units (Scheme 1). The solid substrate chosen for the grafting is a silicon wafer because it is atomically smooth, partly transparent to infrared wavelengths, and reflective for the UV–visible wavelengths. This wafer is well adapted for multiple characterization techniques such as ellipsometry, contact angle, and infrared spectroscopy. The thickness of the COS layer was determined by ellipsometry and the surfaces were characterized by attenuated total reflectance infrared spectroscopy (ATR-FTIR), contact angle measurements, atomic force microscopy (AFM), and time-of-flight secondary ion mass spectrometry (ToF-SIMS). Finally, the grafting was also extended to chitosan oligomers of higher DA.

## 2. Results and Discussion

### 2.1. Synthesis and Characterization of Amf-Terminated Chitosan Oligomers (COSamf)

**Scheme 2:**
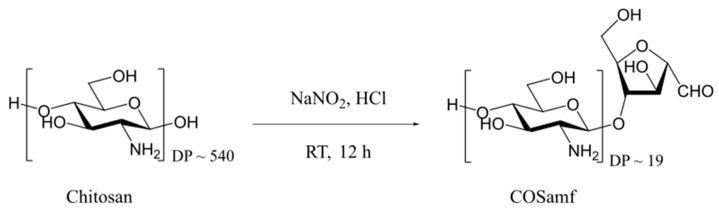
Synthesis of COSamf by nitrous acid depolymerization of fully *N*-deacetylated chitosan.

Amf-terminated chitosan oligomers (COSamf) with well-defined structural parameters (average DP ~19 and DA ~0%) were synthesized from fully *N*-deacetylated chitosan by nitrous acid depolymerization (Scheme 2). The nitrous acid depolymerization of chitosan is a well-known method to obtain chitosan oligomers with controlled DPs [25,28,29,30]. COSamf was prepared according to a process detailed in our previous studies [23,25,27,31]. Briefly, the nitrous acid depolymerization was performed at room temperature in a homogeneous dilute aqueous acid solution of chitosan by adding a specific amount of NaNO_2_. Indeed, the number of glycosidic bonds broken is roughly stoichiometric to the amount of added NaNO_2_. This depolymerization mechanism involves the reaction of nitrous acid with the amine group of the GlcN unit and leads to a 2,5-anhydro-d-mannofuranose reducing-end unit (amf).

**Figure 1:**
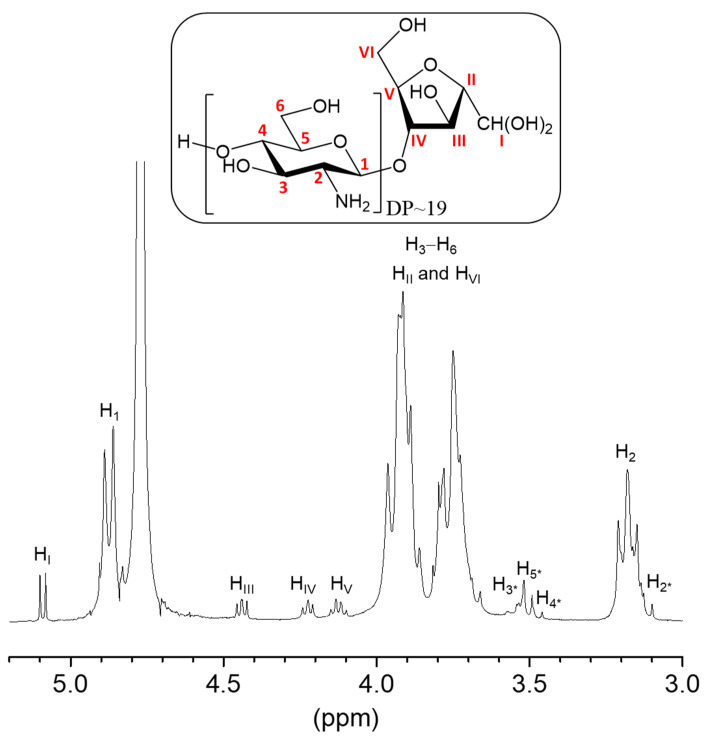
^1^H NMR spectrum (300 MHz) of COSamf (average DP ~19, DA ~0%) in D_2_O/HCl at 300 K. Note that the *gem*-diol hydrated form of the amf-unit aldehyde was the only form detected [25]. H atoms of the non-reducing end GlcN unit are indicated with an asterisk.

The chemical structure of COSamf was confirmed by ^1^H NMR spectroscopy and the corresponding spectrum (Figure 1) corroborates those obtained through the same depolymerization process by Moussa et al. [25] and Coudurier et al. [23]. It is worth noting that the *gem*-diol hydrated form -CH(OH)_2_ of the amf-unit aldehyde was the only form detected using ^1^H NMR analysis in D_2_O/HCl at room temperature. The average GlcN repeating unit number, i.e., the DP, was determined by ^1^H NMR spectroscopy from the relative peak areas of the proton H_I_ of the amf unit (${A_{H}}_{I}\left(amf\right)$) at 5.10 ppm and the proton H_2_ of the GlcN unit (${A_{H}}_{2}\left(GlcN\right)$) at 3.20 ppm according to Equation (1):

(1) $$DP=\frac{{A_{H}}_{2}\left(GlcN\right)}{{A_{H}}_{I}\left(amf\right)}$$

The molecular parameters of COSamf were also determined by SEC analysis (see Table S1 in Supporting Information), according to an analytical method previously detailed by Schatz et al. [32], and they agreed with the average DP of ~19 calculated from ^1^H NMR results. Moreover, TGA analysis showed a low water content within the powder (<3% *w*/*w*) and a degradation temperature starting around 200 °C (see Figure S1 in Supporting Information). The production of COSamf with such DP was chosen due to the easy isolation and purification of the product by simple precipitation in basic conditions.

### 2.2. Reductive Amination of COSamf with 3-Methoxyaniline

**Scheme 3:**
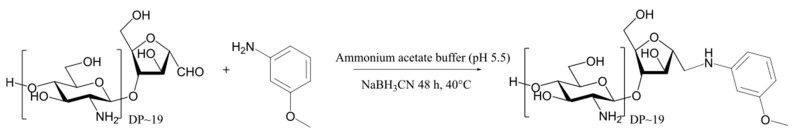
Reducing-end grafting of COSamf with 3-methoxyaniline by reductive amination.

Chitosan oligomers with a 2,5-anhydro-d-mannofuranose (amf) unit at the reducing end can be further used for the regiospecific grafting of COS brushes onto a solid substrate via the amf-unit aldehyde. In order to determine the appropriate reductive amination conditions for the grafting of amf-unit aldehydes with *m*-aminophenoxy-modified wafers, a model reaction was carried out with 3-methoxyaniline in a homogeneous medium (Scheme 3). This aromatic amine was selected due to its low pKa value (~4.2) compared to the one of chitosan oligomers (pKa 6.5–7) [25]. The amine function of the 3-methoxyaniline is then supposed to be more nucleophilic than the amine functions of COSamf in the slightly acidic aqueous conditions (pH 5.5) which are needed for the solubilization of chitosan oligomers.

**Figure 2:**
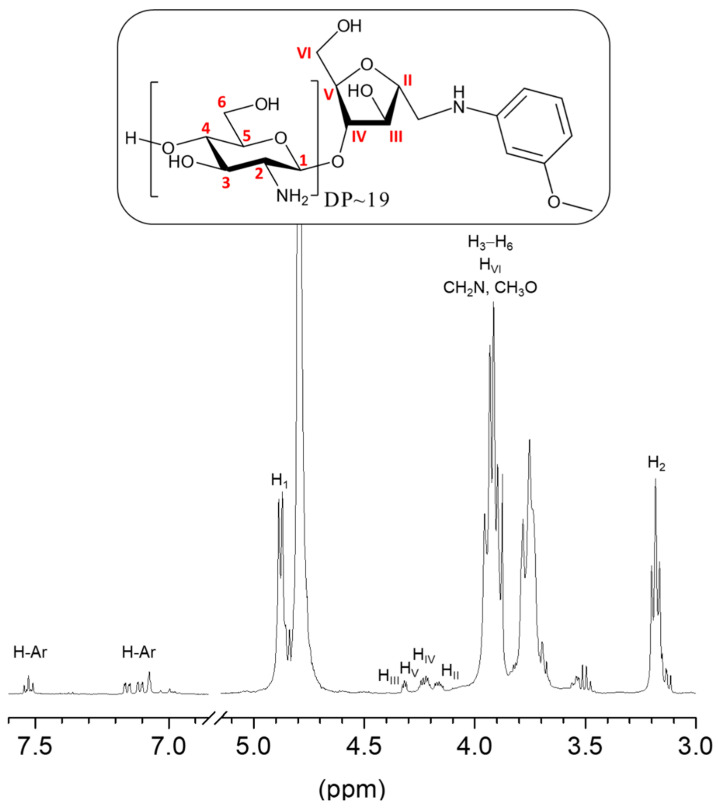
^1^H NMR spectrum (300 MHz) of 3-methoxyaniline-linked COSamf in D_2_O/HCl at 300 K.

The reductive amination of COSamf using an excess of 3-methoxyaniline (10 eq./amf unit) was carried out in ammonium acetate buffer (0.15 M, pH 5.5) at 40 °C for 2 days in the presence of a reducing agent (NaBH_3_CN), based on reaction conditions described by Moussa et al. [25]. 3-Methoxyaniline-linked COSamf was then precipitated in alkaline conditions by the addition of NH_4_OH solution. A white powder with a mass yield of 81% was obtained. ^1^H NMR (Figure 2) and COSY 2D NMR analyses (see Figure S2 in Supporting Information) confirmed the expected structure of the modified COSamf. Indeed, the functionalization was proven by the apparition of the CH_2_N signal at 3.8 ppm which is consistent with the NMR spectra obtained by Moussa et al. [25] for COSamf modified with other aromatic amine derivatives. In addition, aromatic signals between 7 and 7.6 ppm gave access to the degree of functionalization of COSamf by comparison with the intensity of the proton H_2_ of the GlcN unit, indicating that the functionalization reaction was quantitative. Moreover, the average DP, calculated using Equation (1) and replacing the proton H_I_ of the amf unit (${A_{H}}_{I}\left(amf\right)$) by the proton H_III_ of the same unit (${A_{H}}_{III}\left(amf\right)$), was preserved after the COSamf functionalization (DP ~19).

### 2.3. Grafting of COSamf onto M-Aminophenoxy-Modified Silicon Wafers

#### 2.3.1. Silanization of Silicon Wafers with APPTMS

The first step consisted in obtaining a silane monolayer by immersing silicon wafers into a 1% *v*/*v* APPTMS solution in dry toluene (Scheme 1). Silane coupling agents are commonly used to functionalize surfaces for subsequent polymer graftings [33]. The APPTMS was selected due to its chemical structure, similar to the 3-methoxyaniline model compound and thus probably equally reactive towards the COS reducing end. Indeed, it is an aromatic amine silane which should be more nucleophilic and thus more reactive than the amine functions of chitosan oligomers in slightly acidic aqueous conditions (pH 5.5). The morphology and the homogeneity of silanized surfaces are important for controlling the upcoming chitosan oligomers grafting. The modification reaction was carried out by exposing the silicon substrate to an APPTMS solution for various times (1 h to 6 h) to optimize the thickness of the silanized layer. Assuming that all bonds have lengths close to those of carbon–carbon bonds, the theoretical thickness corresponding to the maximum grafting density for a monolayer can be estimated to 10 Å. The thickness of the layer and contact angles increased when the exposure time increased (see Figure S3 in Supporting Information). A silanization time of 5 h appeared to lead to a dense APPTMS monolayer (grafting thickness of 1.0 nm) with limited reticulation of the trifunctional silane and the obtained surfaces were smooth (see AFM images in Figure S4 and corresponding roughness data in Table S2 in Supporting Information).

#### 2.3.2. Grafting of COSamf Brushes Onto Silicon Wafers

(a) Optimization of the grafting process using ATR-FTIR

**Figure 3:**
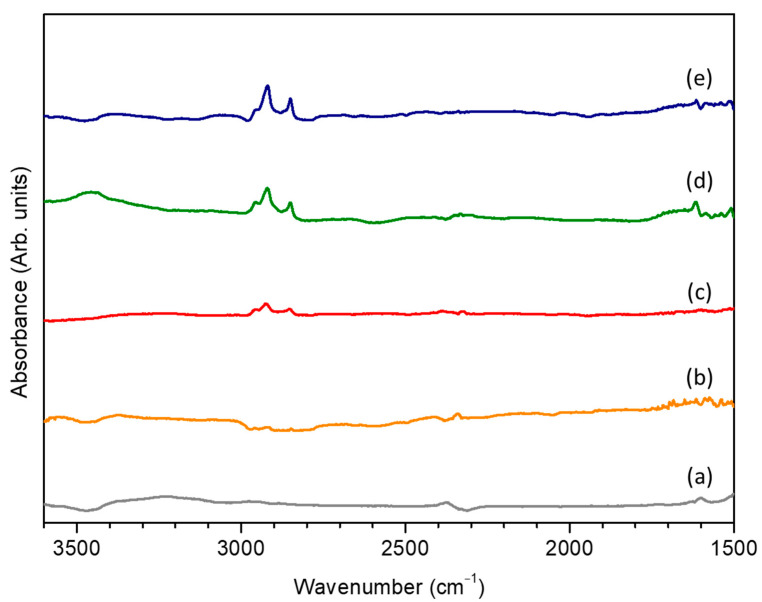
ATR-FTIR spectra of silicon crystals at various steps of the grafting process: (**a**) crystals silanized for 5 h in dry toluene, then immersed in the COSamf solution (3% *w*/*v*) at 40 °C and pH 5.5: (**b**) for 5 min without NaBH_3_CN reduction, (**c**) for 1 h, then 2 days of reduction, (**d**) for 3 days, then 3 h of reduction, and (**e**) for 3 days without reduction. Curves were vertically shifted for the sake of clarity.

In this study, temperature and pH conditions used for the coupling of COSamf with 3-methoxyaniline (see Section 2.2) were applied to optimize the grafting of COSamf brushes onto *m*-aminophenoxy-modified silicon wafers. Thus, the silicon surfaces were immersed in a 3% *w*/*v* COSamf solution at 40 °C and pH 5.5 at various times. The addition of NaBH_3_CN to reduce the imine functions was also studied in order to increase the stability of the grafting bonds to hydrolysis. Indeed, the intrinsic hydrolyzable character of imine can lead to the removal of the brushes from the surface. The grafting was carried out in an acidic aqueous solution (pH = 5.5) where the amine functions of APPTMS would be mostly neutralized and thus more reactive with the amf-unit aldehyde, whereas the amine functions of COSamf would be mostly protonated, thus less reactive. The reaction could not be carried out at higher pH, due to the low solubility of COSamf in neutral aqueous solutions. The grafting of the COSamf brushes was monitored by ATR-FTIR spectroscopy on silicon crystals. The influence of the reaction time and that of the imine reduction were investigated and the corresponding IR spectra are reported in Figure 3.

The silane monolayer was too thin to be detected by ATR-FTIR (Figure 3a), while the grafting reaction performed using the conditions of the model reaction (see Section 2.2), i.e., during 1 h then 2 days of NaBH_3_CN reduction, showed a small signal around 2900 cm^−1^ corresponding to the C-H stretching bonds of chitosan oligomers (Figure 3c). The intensity of the signal and thus the amount of grafted brushes were higher when the reaction was carried out for 3 days, independently of the addition of NaBH_3_CN (Figure 3d or Figure 3e). The *m*-aminophenoxy-modified wafers were also immersed only for 5 min in the COSamf solution to evaluate the adsorption amount of the brushes (Figure 3b). It showed no signal in ATR-FTIR which proved the successful modification of the surface when the reaction was carried out for 3 days. As expected, the heterogenous grafting reaction on a solid substrate is more sluggish than in a homogeneous solution (see Section 2.2) which explains the longer reaction time required for the grafting of the COSamf onto the silicon wafers. Hence, for further characterizations, only chitosan oligomers brushes grafted at 40 °C and pH 5.5 for 3 days, with and without the imine reduction using NaBH_3_CN were selected.

(b) Physicochemical characterization of the grafted COSamf brushes

The dry thickness of the chitosan oligomer brushes was measured by ellipsometry. Unlike the reference wafer (i.e., a silanized wafer immersed in the COSamf solution at 40 °C for 5 min) for which all chitosan oligomers were removed after ultrasonication and acidic washing, the wafers grafted for 3 days exhibited a COS layer thickness of a few nanometers. Interestingly, the brushes grafted for 3 days with NaBH_3_CN reduction exhibited a COS layer thickness of 3.4 ± 0.2 nm versus 2.7 ± 0.1 nm for the ones obtained without NaBH_3_CN reduction. Moreover, grafted wafers obtained with and without NaBH_3_CN reduction gave contact angle values of 75.8° ± 2.0° and 72.7° ± 2.7°, respectively. This result is consistent with the contact angles measured on low DA chitosan-coated surfaces (76.5° ± 2.1° according to [34]). For comparison, the contact angle measured for the reference wafer was 61.1° ± 0.5°, this angle value being similar to the one measured for the silanized wafer (59.9° ± 0.7°). These data suggest that the addition of the reduction step increased the COS grafting density and/or led to a covalent grafting less sensitive to hydrolysis.

**Figure 4:**
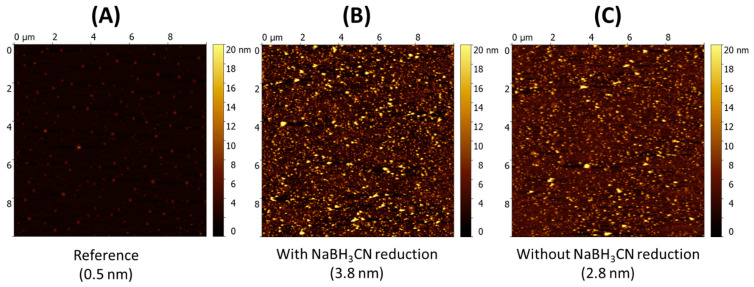
AFM images and RMS roughness data (in brackets) of chitosan oligomers brushes grafted on silicon wafers. Wafers after immersion in the COSamf solution (3% *w*/*v*) at 40 °C and pH 5.5 for: (**A**) 5 min (i.e., reference wafer), (**B**) for 3 days with NaBH_3_CN reduction, and (**C**) for 3 days without NaBH_3_CN reduction.

The topology of the brushes was investigated by AFM and the roughness was measured by the root-mean-square deviation (RMS) of the surface (Figure 4). The topology of the *m*-aminophenoxy-modified surface suggested a slight reticulation of the trifunctional silane at the surface. The reference wafer exhibited a roughness comparable to that of the silanized surface. On the contrary, the grafted wafers exhibited much higher RMS roughness values. This result highlights the successful grafting of chitosan oligomers brushes on silicon wafers via their reducing ends, with or without the addition of the reduction step.

The COS-grafted wafers were also characterized by ToF-SIMS. ToF-SIMS allows the identification of molecular structures at the extreme surface of materials (information depth limited to a few monolayers). The ToF-SIMS positive mode spectra of both the piranha washed surface and the silanized surface were compared (see Figures S5 and S6, respectively, in Supporting Information). The piranha-washed surface is as expected characterized by a strong relative intensity for Si^+^ (27.97 *m/z*) but also by the detection of polydimethylsiloxane (PDMS) signatures (contamination). The silanized surface also exhibits Si^+^ (27.97 *m/z*) as well as some contamination (PDMS and a nitrogen-containing contamination). However, some signatures specific to the aromatic part of the silane, such as C_6_H_5_^+^ (77.04 *m/z*) and C_7_H_7_^+^ (91.05 *m/z*), were specifically detected in the spectrum of the silanized surface.

Then, the COSamf powder was analyzed to identify its specific ToF-SIMS signature and thus facilitate the interpretation of the spectra for the surfaces grafted with COSamf. The ToF-SIMS positive mode spectrum of the COSamf powder showed signatures specific to the COSamf molecule, such as the peaks at *m/z* = 112.04 (C_5_H_6_NO_2_^+^), 144.07 (C_6_H_10_NO_3_^+^) as well as that at *m/z* = 162.08 (C_6_H_12_NO_4_^+^) corresponding to the ion of the GlcN repeating unit of COSamf (see Figure S7 in Supporting Information). These peaks were also found in the spectra of the COSamf brushes grafted onto the surface (see Figures S8 and S9 in Supporting Information), but with a very small relative intensity. Signals at low *m/z* were observed in both spectra and can be attributed to C_x_H_y_NO^+^ and C_x_H_y_O_z_^+^ type ions: C_2_H_4_NO^+^ (*m/z*= 58.03), C_2_H_6_NO^+^ (*m/z* = 60.04), C_3_H_4_NO^+^ (*m/z* = 70.03) and C_5_H_5_O_2_^+^ (*m/z* = 97.03). However, these ions were again detected with a very small relative intensity. The characteristic peaks of the silicon wafer were significantly less detected on the ToF-SIMS positive mode spectrum of the COS-grafted wafers compared to the case of the silanized surface. This confirms the detection of material at their surface. However, a small relative intensity for signatures related to the COSamf molecule may indicate some unspecific extreme surface contamination. Finally, the comparison of ToF-SIMS spectra in the positive mode of the grafted surfaces obtained with or without the addition of the reduction step (NaBH_3_CN) showed that they present strong similarities (see Figures S8 and S9 in Supporting Information).

In this work, the grafting of chitosan oligomers brushes was also extended to partially acetylated COSamf (paCOSamf). First, fully *N*-deacetylated chitosan was purified and homogeneously *N*-acetylated to a DA of ~21% according to the acetylation method described by Vachoud et al. [35]. paCOSamf was then produced from the acetylated chitosan using the nitrous acid depolymerization process explained in Section 3.2.1 for fully *N*-deacetylated chitosan (see also the experimental method detailed in Supporting Information). The amount of NaNO_2_ added was calculated for a GlcN unit/NaNO_2_ molar ratio equal to 8. In this depolymerization process, paCOSamf was partly soluble in alkaline solutions. Thus, they were not precipitated by the addition of an NH_4_OH solution, but the acidic aqueous solution of paCOSamf was directly freeze-dried. Indeed, as shown by Tommeraas et al. [30], COSamf can be degraded when freeze-dried in alkaline conditions leading to the formation of 5-hydroxymethylfurfural (HMF). The ^1^H NMR analysis of the freeze-dried product (see Figure S10 in Supporting Information) confirmed that paCOSamf with an average DP around 17 and a preserved DA of ~21% was obtained. Without further purification, paCOSamf was then grafted onto *m*-aminophenoxy-modified silicon wafers as described in Section 3.3 using the following grafting conditions: 3 days of reaction at 40 °C without the addition of the reduction step. Due to the better solubility of these oligomers in neutral aqueous solutions, the pH of the paCOSamf solution could be increased to 6.5 to favor grafting. The surfaces gave a contact angle value of 52.1° ± 2.7° and a COS layer thickness of 2.2 ±0.5 nm, which proved the successful grafting of COSamf of DA ~21%.

## 3. Materials and Methods

### 3.1. Materials

Chitosan from shrimp shells was provided by Mahtani Chitosan from batch type 244 with a DA of ~0.5% determined by ^1^H NMR, a mass-average molar mass (*M*_w_) of 196 kg.mol^−1^, and a dispersity (*Đ*) of 2.27 determined by size-exclusion chromatography. TGA analysis (SDT Q600, TA Instruments, 2 °C.min^−1^ up to 200 °C then 20 °C.min^−1^ up to 900 °C, 50 min isotherm, air) showed that the chitosan powder contained 9.5% of water. Hydrochloric acid (HCl, 37% *w*/*w*), sodium nitrite (NaNO_2_, ≥99%), sodium cyanoborohydride (NaBH_3_CN, >95%), 3-methoxyaniline (97%), acetic acid (AcOH, >99.9% *w*/*w*), hydrogen peroxide (H_2_O_2_, 40% *w*/*w*), sulfuric acid (H_2_SO_4_, 96% *w*/*w*), ammonium hydroxide (NH_4_OH, 28% *w/w*), toluene, anhydrous toluene, and acetic anhydride (>99%) were purchased from Sigma-Aldrich (Merck KGaA, Darmstadt, Germany). 3-(*m*-aminophenoxy)propyltrimethoxysilane (APPTMS, ≥98%) was purchased from ABCR (Karlsruhe, Germany), 2-propanediol (99.5%) from CARLO ERBA Reagents (Val-de-Reuil, France) and water was obtained from the Simplicity^®^ Milli-Q water purification system providing ultrapure water (resistivity of 18.2 MΩ·cm). Silicon wafers (doped-P bore, orientation (100)) were purchased from Siltronix^®^ (Archamps, France).

### 3.2. Synthesis and Characterization of Amf-Terminated Chitosan Oligomers (COSamf)

#### 3.2.1. Synthesis of COSamf (Average DP ~19 and DA ~0%)

Chitosan (15 g, 83.8 mmol of GlcN unit) was dissolved in 500 mL of deionized water with the addition of 7.6 mL HCl (37% *w*/*w*). Twelve milliliters of a freshly prepared aqueous solution of NaNO_2_ (723 mg, 10.5 mmol for a GlcN unit/NaNO_2_ molar ratio equal to 8) was added and the reaction was allowed to proceed for 12 h at room temperature. The solution was filtered through a cellulose membrane (1.2 µm) and the chitosan oligomers were then precipitated by the addition of an NH_4_OH solution (28% *w*/*w*) to pH 9–10. The precipitate was washed several times by centrifugation with water until neutral pH and was finally freeze-dried. COSamf (9.8 g, 65% mass yield) was obtained as a white powder and stored at −20 °C. ^1^H NMR spectrum was recorded on a Bruker Advance III 300 MHz spectrometer at 300 K according to an analysis method previously described [23]. ^1^H-NMR (300 MHz, D_2_O) of COSamf (Figure 1): δ (ppm) 5.10 (d, *J* = 5.4 Hz, 1H, H_I_), 4.90–4.80 (m, 19H, H_1_), 4.45 (t, *J* = 4.9 Hz, 1H, H_III_), 4.23 (t, *J* = 4.9 Hz, 1H, H_IV_), 4.13 (m, 1H, H_V_), 4.05–3.45 (m, 98H, H_II_ and H_VI_, H_3_ to H_6_), 3.25–3.08 (m, 19H, H_2_).

#### 3.2.2. Reductive Amination of COSamf with 3-Methoxyaniline

COSamf was end-functionalized with 3-methoxyaniline (Scheme 3) according to a protocol previously described by Moussa et al. [25] for the modification of COSamf with functionalized anilines. COSamf (200 mg, 0.06 mmol of amf unit) was solubilized into 6 mL of aqueous ammonium acetate buffer (0.15 M, pH 5.5). 3-methoxyaniline (77 mg, 10 eq./amf unit) in 6 mL of ethanol was added dropwise under stirring. The pH of the solution was adjusted to 5.5 by addition of AcOH and the solution was stirred at room temperature for 60 min. Sodium cyanoborohydride (NaBH_3_CN, 39 mg, 10 eq./amf unit) was added portion-by-portion and the mixture was stirred at 40 °C for 2 days. The oligomers were then precipitated by the addition of an NH_4_OH solution (28% *w*/*w*) to pH 9–10 and washed several times by centrifugation with water/ethanol (1/1, *v*/*v*), then with water until neutral pH. The precipitate was finally freeze-dried leading to 3-methoxyaniline-linked COSamf (171 mg, 81% mass yield) in powder. ^1^H-NMR (300 MHz, D_2_O) of 3-methoxyaniline-linked COSamf (Figure 2): δ (ppm) 7.55–7.49 (m, 1H, H-Ar), 7.20–6.95 (m, 3H, H-Ar), 4.90–4.80 (m, 19H, H_1_), 4.35–4.05 (m, 4H, H_II_ to H_V_), 4.05–3.45 (m, 102H, H_VI_, CH_2_N, CH_3_O, H_3_ to H_6_), 3.25–3.10 (m, 19H, H_2_).

### 3.3. Functionalization and Characterization of Silicon Wafers

The received wafers were cut into squares of ~2 × 2 cm^2^. To clean them from organic pollution and generate the silanol groups at the surface, the silicon wafers were immersed in a piranha bath (H_2_SO_4_/H_2_O_2_, 7/3 *v*/*v*) heated at 150 °C for 15 min, and then rinsed extensively with water. They were then subjected to ultra-sonication in water for 5 min and dried under a flux of clean air. The modification of freshly cleaned wafers was carried out in a 1% *v*/*v* solution of APPTMS in dry toluene under argon for various reaction times (1 h to 6 h). Silanized wafers were then rinsed with toluene and immersed in an ultra-sonication bath to remove physically adsorbed APPTMS before drying.

To chemically graft oligomers onto modified silicon wafers, COSamf was solubilized in an aqueous ammonium acetate buffer (0.15 M, pH 5.5) and the pH of the solution was adjusted to 5.5 by the addition of AcOH. The *m*-aminophenoxy-modified wafers were then immersed in the 3% *w*/*v* COSamf solution at 40 °C for various times to optimize the grafting. Sodium cyanoborohydride (NaBH_3_CN, 10 eq./amf unit) was added portion-by-portion at various reaction times. The grafted wafers were then rinsed with the buffer solution, and further with deionized water before being ultrasonicated and dried.

For the surface characterizations, the thicknesses of the dry surfaces were measured using an ellipsometer (SE-2000, Semilab) at an incident angle of 75°, close to the silicon Brewster angle. Data were then processed using the SEA (spectroscopic ellipsometry analyzer) software. A Cauchy model was used to fit the experimental data (cos*Δ*, tan*Ψ*), in the spectral range of 250 and 800 nm, depending on fits and regression qualities, to evaluate the thickness. The UV parameters *n* and *k* of the native SiO_2_ layer were set to 1.46 and 0.01, respectively, with a fixed thickness of 1.5 nm. The UV parameters *n* and *k* of the APPTMS layer were set to 1.495 and 0.01, respectively, while those of chitosan oligomers were set to 1.53 and 0.002, respectively. For each method, three measurements were performed on each surface at different positions in order to verify its homogeneity.

The surface topology was characterized by atomic force microscopy (AFM) (CSI nano-observer) operated in tapping mode. AFM tips with spring rate close to 3 N/m were purchased from AppNano and images were recorded over an area of 10 µm × 10 µm. The AFM images were processed using the Gwyddion software.

Contact angles were measured using a tensiometer (Easydrop, Kruss) comprising a camera connected to a computer equipped with a drop shape analysis software. To deliver the 1 µL liquid drop on the surface, a 1 mL Hamilton syringe was used, fitted with a needle of 0.5 mm diameter.

FTIR analyses were recorded on a Nicolet iS10 infrared spectrometer operated in the attenuated total reflectance Fourier Transform Infrared Spectroscopy mode (ATR-FTIR). A cooled HeCdTe-based detector was used. A multi-reflection device (Gateway^TM^) purchased from Eurolabo was used with a trapezoidal silicon crystal (specific edge profile cut at 45° angle, with 70 × 10 × 1 mm dimensions, bought from ACM, Villiers Saint Frédéric, France). A homemade accessory was added to make the crystal tailored to the device. A wave number interval ranging from 4000 to 1500 cm^−1^ for a resolution of 4 cm^−1^ and a number of 512 scans were selected to perform the experiment.

Time-of-flight secondary ion mass spectrometry (ToF-SIMS) was recorded on a TRIFT III ToF-SIMS instrument from Physical Electronics operated with a pulsed 22 keV Au^+^ ion gun (ion current of 2 nA) rastered over a 300 µm × 300 µm surface area. An electron gun was operated in pulsed mode at low electron energy for charge compensation. Data were then processed using the WinCadence^TM^ software and hydrocarbon secondary ions were selected for mass calibration.

## 4. Conclusions

An innovative process for the functionalization of silicon wafers using COS brushes was successfully developed. This process involved a regiospecific chemical grafting by reductive amination of the reducing-end aldehydes of chitosan oligomers with aromatic amines of silanized silicon wafers. First, fully *N*-deacetylated chitosan oligomers with an average number of GlcN repeating units of 19 and bearing a 2,5-anhydro-d-mannofuranose (amf) unit at the reducing end were prepared by nitrous acid depolymerization of fully *N*-deacetylated chitosan. Then, the reactivity of the amf-unit aldehydes to aromatic amines was first studied in a homogeneous medium. A model reaction was performed in acidic aqueous solutions between COSamf and 3-methoxyaniline in reductive amination conditions. NMR analyses of the reaction product confirmed the chemical structure of the expected 3-methoxyaniline-linked chitosan oligomers and the efficiency of the developed coupling conditions. After a silanization step where silicon wafers were modified with 3-(*m*-aminophenoxy)propyltrimethoxysilane (APPTMS), chitosan oligomers were successfully grafted to the *m*-aminophenoxy-modified wafers via their reducing ends leading to COS brushes grafted surfaces. Smooth surfaces were obtained with a COS layer thickness of a few nanometers. It was shown that the addition of the reduction step (NaBH_3_CN) could positively improve the COS grafting density and/or lead to a covalent grafting less sensitive to hydrolysis. Finally, this grafting process was also extended to chitosan oligomers with a higher DA (i.e., ~21%). In this case, partially acetylated COSamf was produced by nitrous acid depolymerization of chitosan of DA ~21%. Then, it was successfully grafted to *m*-aminophenoxy-modified silicon wafers leading to partially acetylated chitosan oligomers brushes with a layer thickness of a few nanometers.

## Data Availability

Not applicable.

## Supplementary Materials

The supporting information can be downloaded at: https://www.mdpi.com/article/10.3390/ijms23148013/s1. References are cited in [25,35,36,37].
