# Supplementary material for: Regiospecific Grafting of Chitosan Oligomers Brushes onto Silicon Wafers
Source: Int J Mol Sci. 2022 Jul 20;23(14):8013. doi: 10.3390/ijms23148013 (PMC9322837; doi:10.3390/ijms23148013)
Supplement: Supplementary file 1 [file ijms-23-08013-s001.zip › ijms-1798065-supplementary.pdf]

Regiospecific grafting of chitosan oligomers brushes onto silicon wafers

Cyrielle Garreau [1], Corinne Gablin [2], Didier Léonard [2], Thierry Delair [1], Guillaume Sudre [1] and Stéphane Trombotto [1,*]

[1] Univ Lyon, CNRS, UMR 5223, Ingénierie des Matériaux Polymères, Université Claude Bernard Lyon 1, INSA Lyon, Université Jean Monnet, F-69622 Villeurbanne, France; garreau.cyrielle@gmail.com (C.G.); thierry.delair@univ-lyon1.fr (T.D.); guillaume.sudre@univ-lyon1.fr (G.S.)
[2] Univ Lyon, CNRS, Université Claude Bernard Lyon 1, Institut des Sciences Analytiques, UMR 5280, 5, rue de la Doua, F-69100 Villeurbanne, France; corinne.gablin@isa-lyon.fr (C.G.); didier.leonard@univ-lyon1.fr (D.L.)

* Correspondence: stephane.trombotto@univ-lyon1.fr

**A. Structural characterization of COSamf (DP ~19, DA ~0%)**	p. 3
**Table S1.** Molecular parameters determined by SEC analysis of COSamf (DP ~19, DA ~0%)	p. 3
**Figure S1.** Thermogravimetric analysis of COSamf (DP ~19, DA ~0%)	p. 3
**B. Reductive amination of COSamf (DP ~19, DA ~0%) with 3-methoxyaniline**	p. 4
**Figure S2.** COSY 2D NMR spectrum (400 MHz) of 3-methoxyaniline-linked COSamf in D_2O/HCl at 300 K	p. 4
**C. Silanization of silicon wafers with APPTMS**	p. 5
**Figure S3.** (A) Thickness and (B) contact angles of silicon wafers functionalized with APPTMS for various reaction times	p. 5
**Figure S4.** AFM images of silicon wafers functionalized with APPTMS for different reaction times from 1 h to 6 h	p. 6
**Table S2.** RMS roughness of silicon wafers functionalized with APPTMS for different reaction times from 1 h to 6 h	p. 6
**D. ToF-SIMS analyses of surfaces**	p. 7

**Figure S5.** Piranha washed silicon wafers: positive ion mode ToF-SIMS spectra in the mass ranges m/z = 25-65; 65-100; 100-150 and 150-200	p. 7
**Figure S6.** *m*-Aminophenoxy-modified silicon wafers: positive ion mode ToF-SIMS spectra in the mass ranges m/z = 25-65; 65-100; 100-150 and 150-200	p. 8
**Figure S7.** COSamf powder: positive ion mode ToF-SIMS spectra in the mass ranges m/z = 0-100; 100-150 and 150-200	p. 9
**Figure S8.** COSamf grafted on *m*-aminophenoxy-modified silicon wafers with $NaBH_3CN$ reduction: positive ion mode ToF-SIMS spectra in the mass ranges m/z = 0-100; 100-150 and 150-200	p. 10
**Figure S9.** COSamf grafted on *m*-aminophenoxy-modified silicon wafers without $NaBH_3CN$ reduction: positive ion mode ToF-SIMS spectra in the mass ranges m/z = 0-100; 100-150 and 150-200	p. 11
**E. Synthesis and characterization of partially acetylated COSamf (DP ~17, DA ~21%)**	p. 12
**Figure S10.** ^1H NMR spectrum of partially acetylated COSamf (DP ~17, DA ~21%) in D_2O/HCl at 300 K	p. 13

A. Structural characterization of COSamf (DP ~19, DA ~0%)

Table S1. Molecular parameters determined by SEC analysis of COSamf (DP ~19, DA ~0%).

Mw ($kg.mol^{-1}$)	Mn ($kg.mol^{-1}$)	Dispersity (Đ)
3.667 ± 0.1	3.151 ± 0.1	1.164 ± 0.05

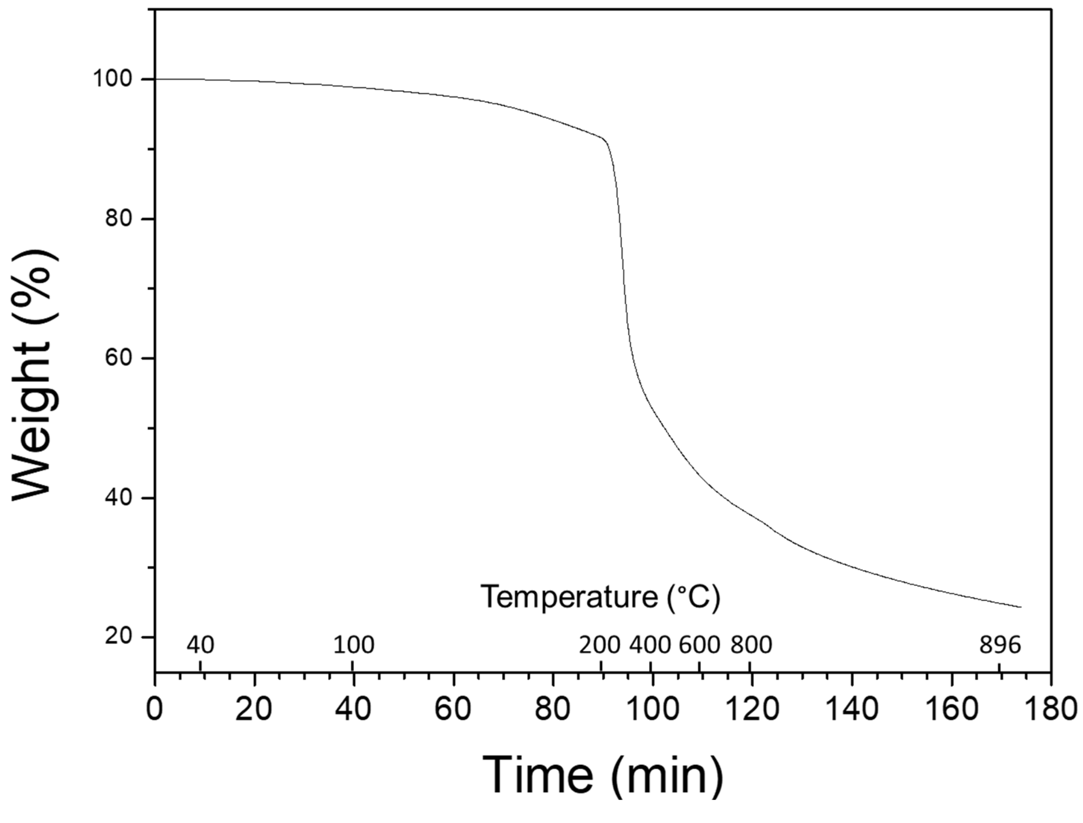

Figure S1. Thermogravimetric analysis of COSamf (DP ~19, DA ~0%).

B. Reductive amination of COSamf (DP ~19, DA ~0%) with 3-methoxyaniline

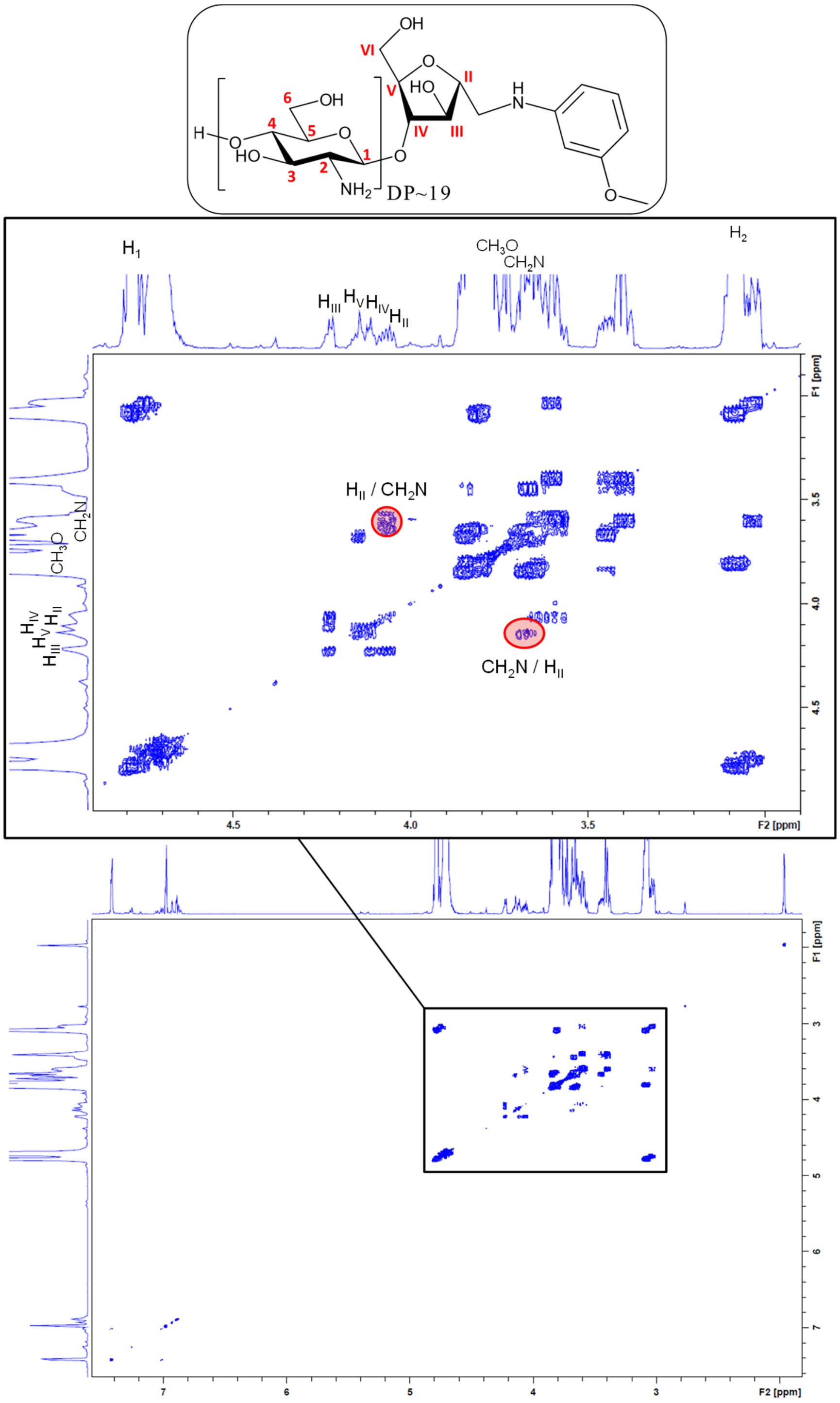

Figure S2. COSY 2D NMR spectrum (400 MHz) of 3-methoxyaniline-linked COSamf in D_2O/HCl at 300 K. It shows the coupling between the proton H_{II} of the amf unit and the protons CH_2N of the 3-methoxyaniline-linked COSamf. The CH_2N signal at 3.8 ppm was indeed hidden behind signals of the GlcN unit (H_3 to H_6) and could not be seen in the ^1H NMR spectrum (Figure 2).

C. Silanization of silicon wafers with APPTMS

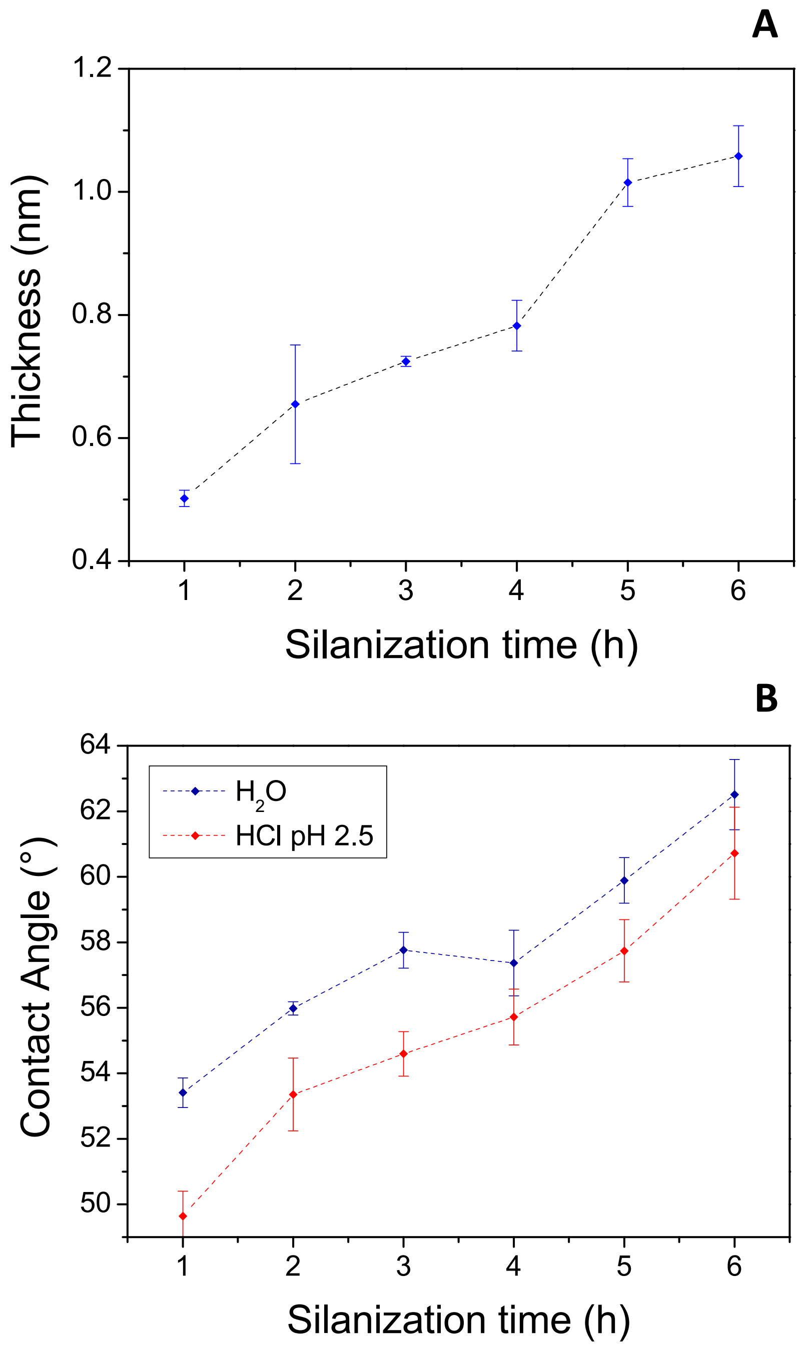

Figure S3. (A) Thickness and (B) contact angles of silicon wafers functionalized with APPTMS for various reaction times. Contact angles were measured with water, but also with an acidic aqueous solution (HCl, pH 2.5) to protonate the amino group of the APPTMS and highlight the wafer silanization.

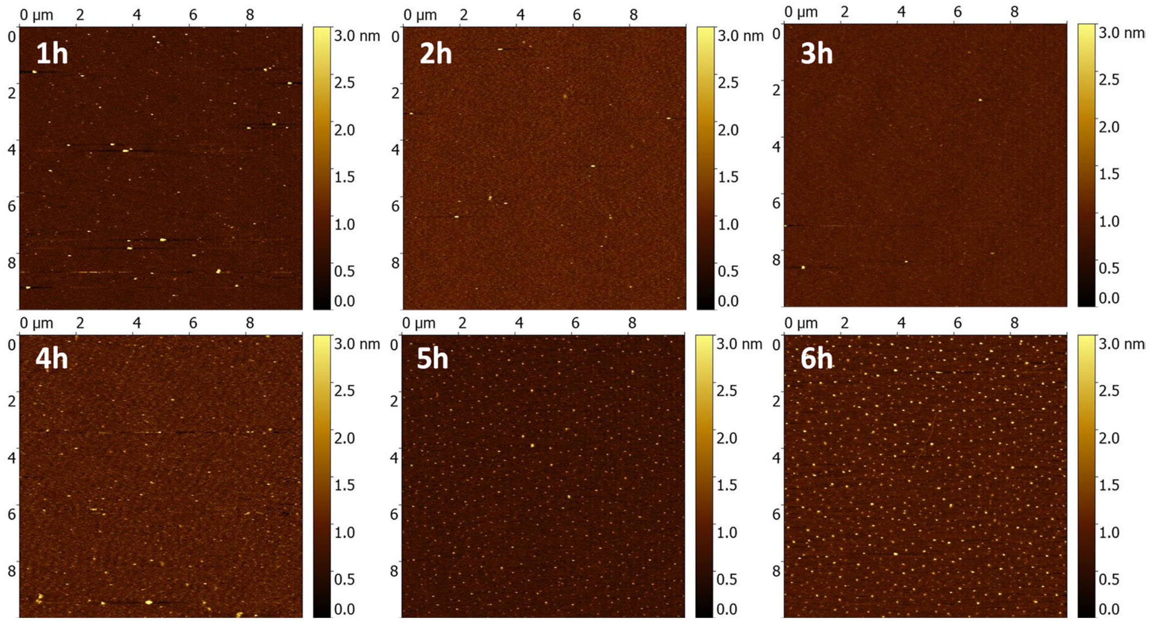

Figure S4. AFM images of silicon wafers functionalized with APPTMS for different reaction times from 1 h to 6 h.

Table S2. RMS roughness of silicon wafers functionalized with APPTMS for different reaction times from 1 h to 6 h.

Silanization time (h)	Roughness (nm)
1	0.177
2	0.158
3	0.121
4	0.217
5	0.195
6	0.265

D. ToF-SIMS analyses of surfaces

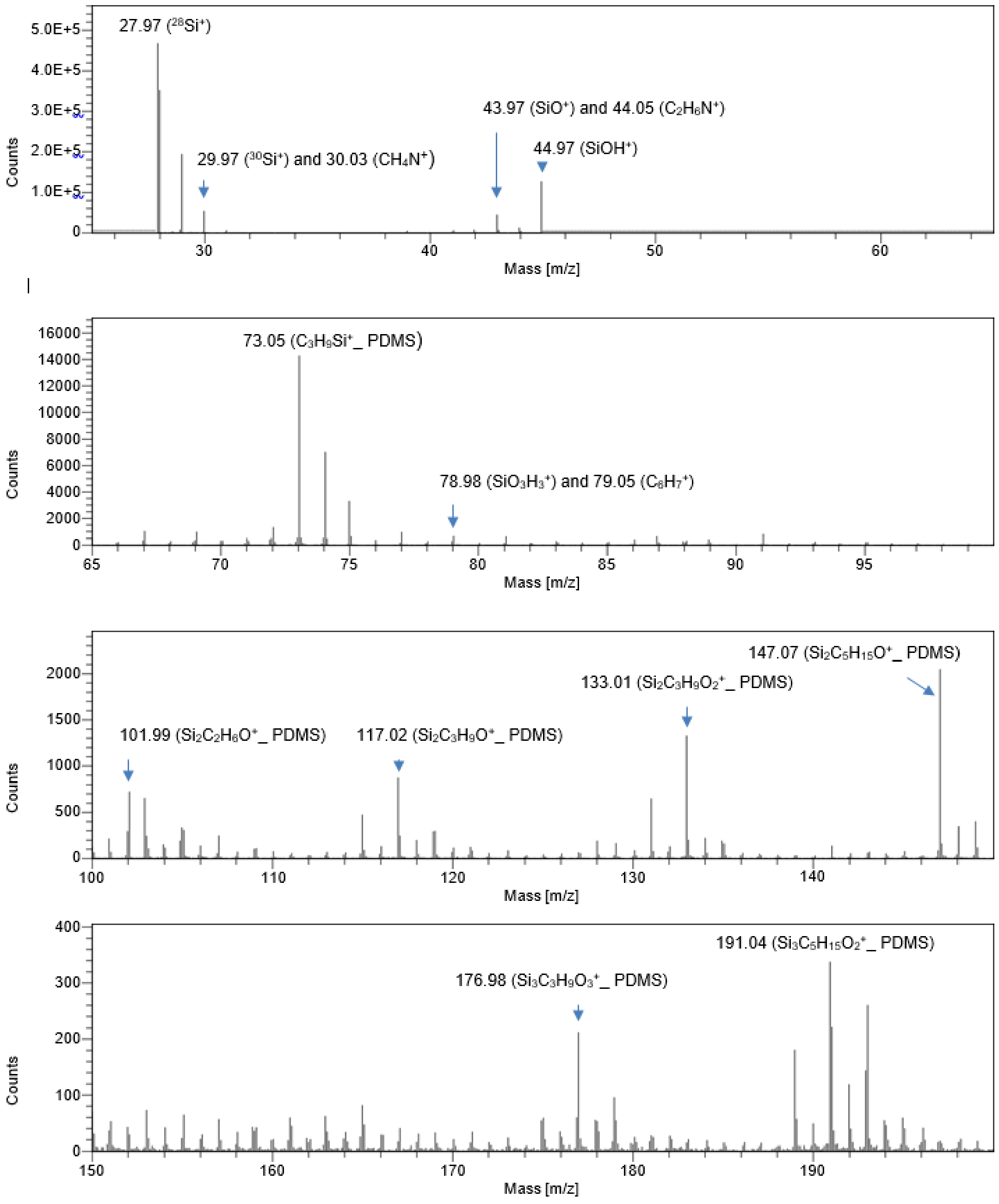

Figure S5. Piranha washed silicon wafers: positive ion mode ToF-SIMS spectra in the mass ranges m/z = 25-65; 65-100; 100-150 and 150-200.

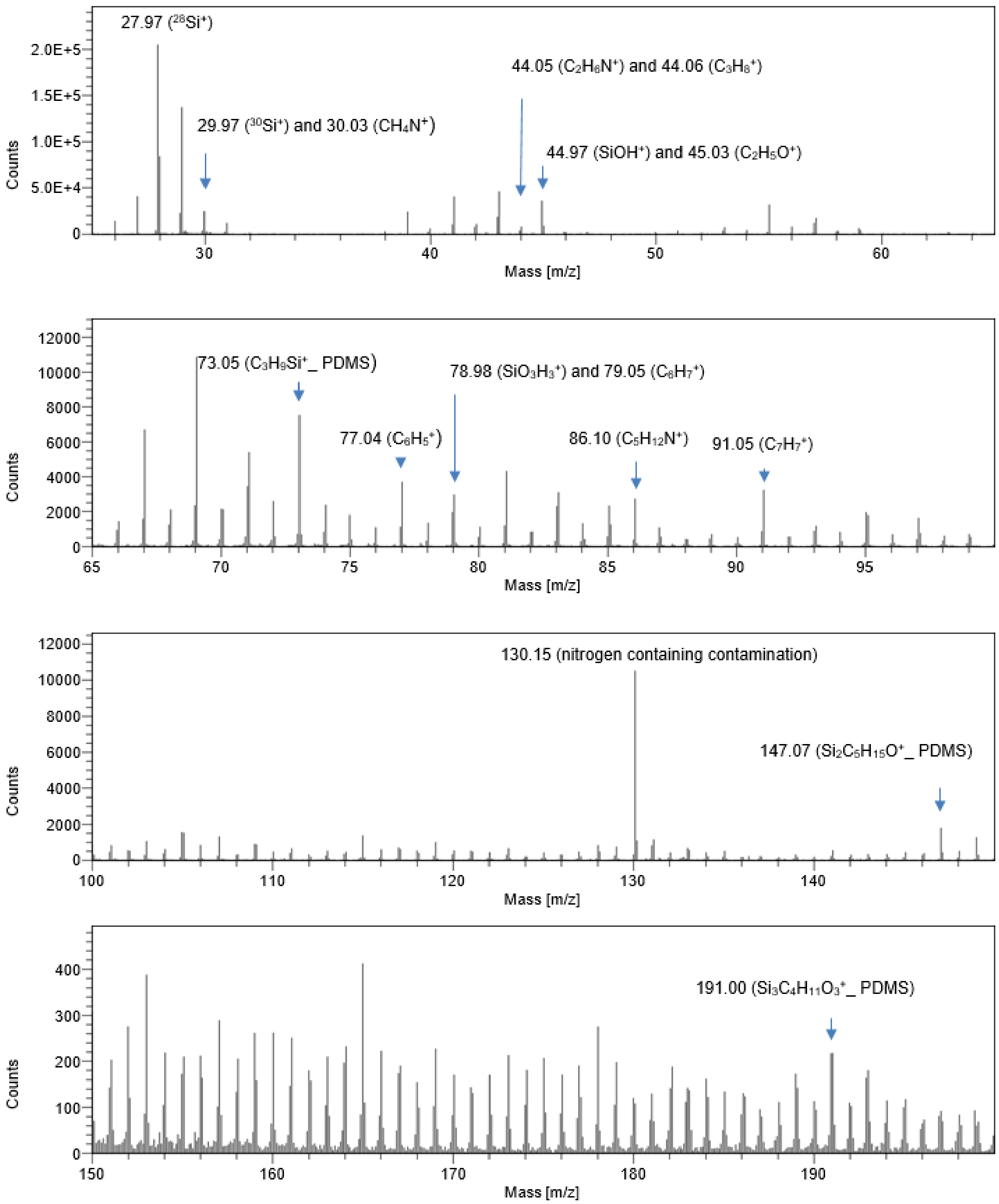

Figure S6. *m*-Aminophenoxy-modified silicon wafers: positive ion mode ToF-SIMS spectra in the mass ranges m/z = 25-65; 65-100; 100-150 and 150-200.

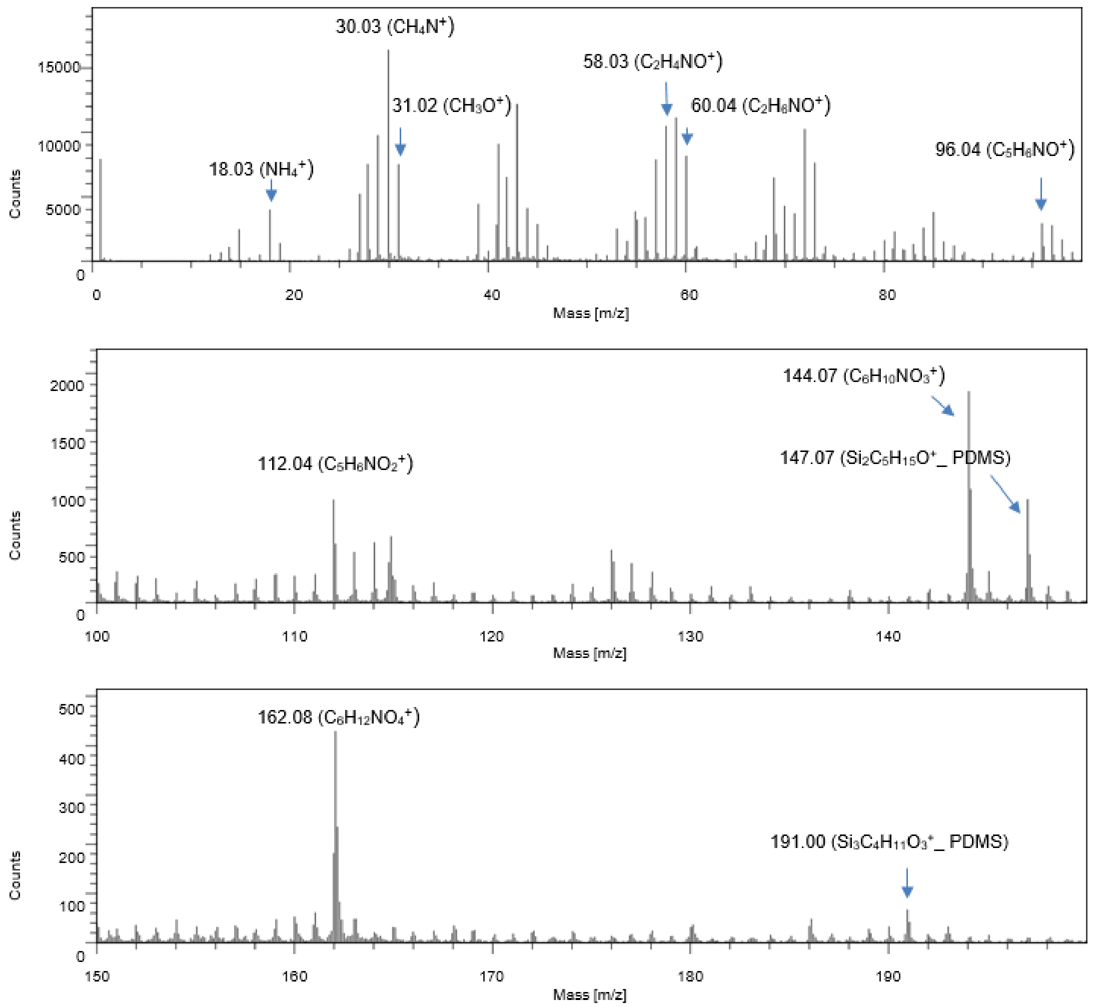

Figure S7. COSamf powder: positive ion mode ToF-SIMS spectra in the mass ranges m/z = 0-100; 100-150 and 150-200.

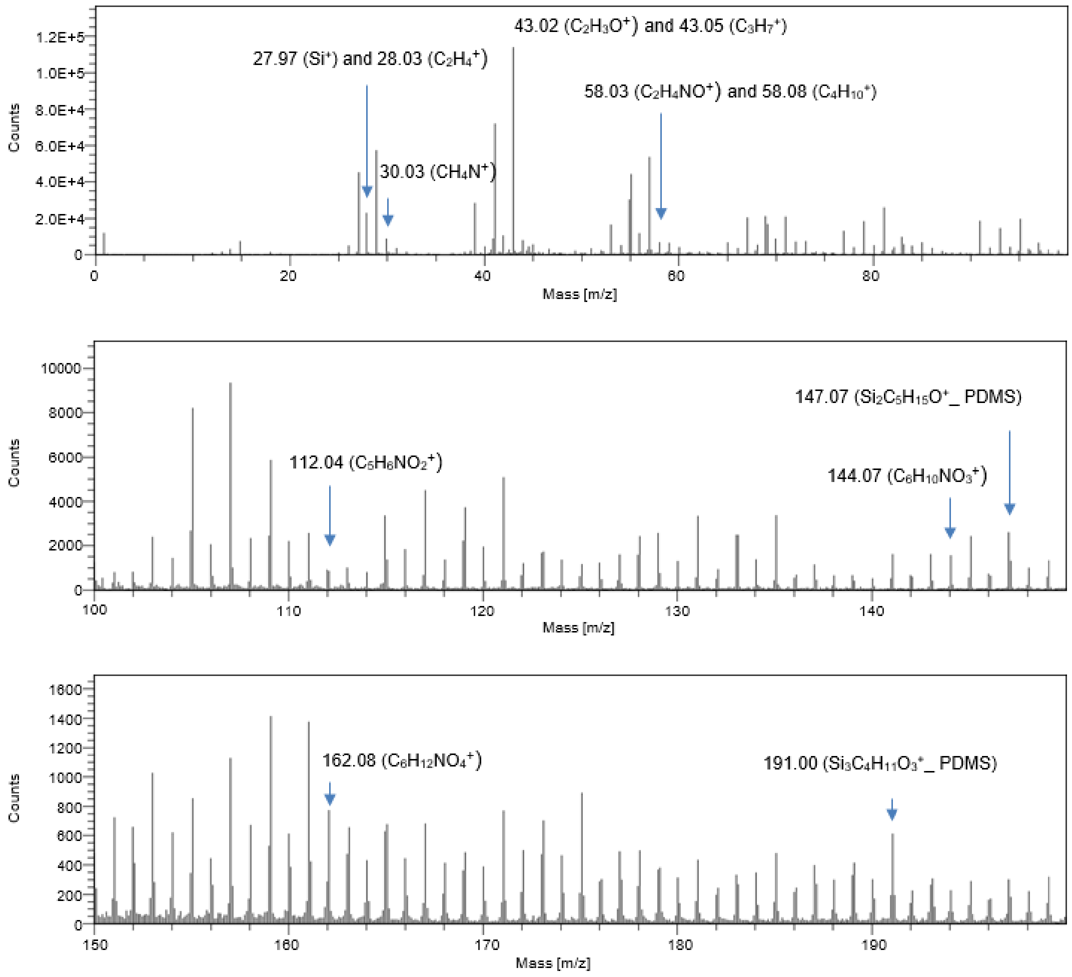

Figure S8. COSamf grafted on *m*-aminophenoxy-modified silicon wafers with $NaBH_3CN$ reduction: positive ion mode ToF-SIMS spectra in the mass ranges m/z = 0-100; 100-150 and 150-200.

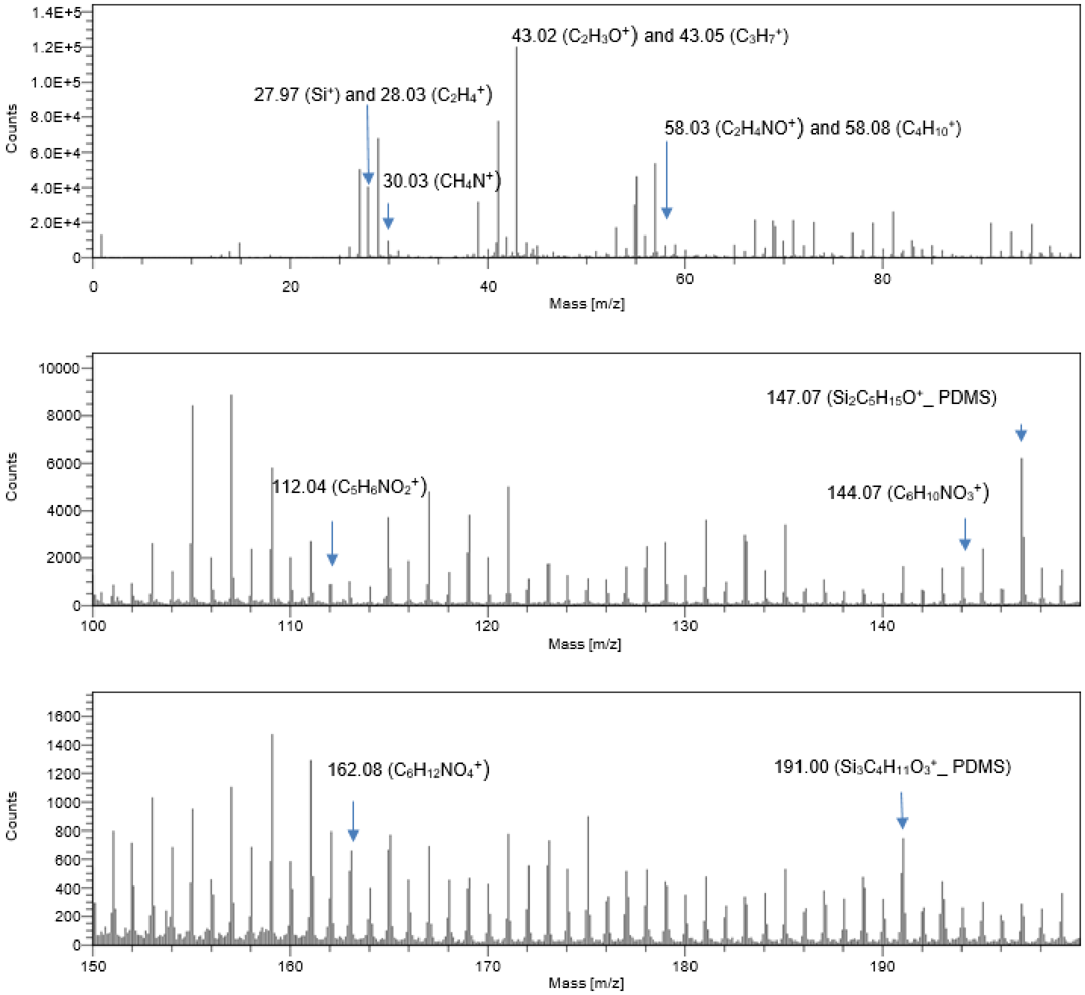

Figure S9. COSamf grafted on *m*-aminophenoxy-modified silicon wafers without $NaBH_3CN$ reduction: positive ion mode ToF-SIMS spectra in the mass ranges m/z = 0-100; 100-150 and 150-200.

E. Synthesis and characterization of partially acetylated COSamf (DP ~17, DA ~21%)

Purification of chitosan: chitosan (DA 0.5%) was first solubilized at 0.5% *w/v* in an AcOH aqueous solution overnight, followed by successive filtrations through cellulose membranes (Millipore®) with decreasing pore sizes from 3 µm to 0.22 µm. Chitosan was then precipitated with NH_4OH and washed several times by centrifugation with Milli-Q water until a neutral pH was obtained. The purified chitosan was finally freeze-dried and stored at room temperature.

Preparation of chitosan of DA ~21%: chitosan (DA ~21%) was prepared according to the *N*-acetylation method previously described by Vachoud *et al.* [35]. Briefly, chitosan (DA ~0.5%) was dissolved in a hydroalcoholic mixture of 1,2-propanediol and AcOH (50/50 v/v). Acetic anhydride was added stoichiometrically to the GlcN unit for 12 h under vigorous mechanical stirring to reach the targeted DA. *N*-acetylated chitosan was precipitated, washed and freeze-dried in the same way as the purification step. The DA of the *N*-acetylated chitosan was calculated by ^{1}H NMR spectroscopy (Bruker Advance III, 300 MHz) according to the method proposed by Hirai *et al.* [36].

Preparation of COSamf (DP ~17, DA ~21%): a 3% *w/v* solution of chitosan (DA ~21%, 1 g, 4.2 mmol of GlcN unit) was prepared in deionized water by addition of 380 µL HCl (37% *w/w*). 1 mL of freshly prepared aqueous solution of $NaNO_2$ (36.3 mg, 0.53 mmol for GlcN/$NaNO_2$ molar ratio = 8) was added and the reaction solution was stirred for 12 h at room temperature. Then, the solution was filtrated through a cellulose membrane (1.2 µm) and freeze-dried. The partially acetylated COSamf (paCOSamf) was obtained as a white powder and stored at -20°C. ^{1}H-NMR (300 MHz, D_2O/HCl) of paCOSamf (Figure S10): δ (ppm) 5.10 (d, J = 5.4 Hz, 0.85H, H_I), 5.00 (d, J = 5.4 Hz, 0.15H, H_{I*}), 4.90-4.80 (m, 13.5H, H_1 (GlcN)), 4.63-4.53 (m, 3.5H, H_1 (GlcNAc)), 4.45 (t, J = 4.9 Hz, 0.85H, H_{III}), 4.35 (t, J = 4.9 Hz, 0.15H, H_{III*}) 4.26-4.02 (m, 2H, H_{IV} H_{IV*} H_V and H_{V*}), 4.02-3.45 (m, 72.7H, H_{II} H_{II*} H_{VI} and H_{VI*}, H_3 to H_6 (GlcN, GlcNAc), H_2 (GlcNAc)), 3.25-3.08 (m, 13.5H, H_2 (GlcN)), 2.02 (s, 10.7H, CH_3 (GlcNAc)). H atoms of the amf unit linked to the GlcNAc unit are indicated with an asterisk.

H_1 to H_6 (GlcNAc) signals were attributed according to Sugiyama *et al.* [37] and the DA was calculated using the equation E1:

$$\mathrm{DA} = \frac{\frac{1}{3}\mathrm{A}_{\mathrm{CH}_3}(\mathrm{GlcNAc})}{\mathrm{A}_{\mathrm{H}_2}(\mathrm{GlcN}) + \frac{1}{3}\mathrm{A}_{\mathrm{CH}_3}(\mathrm{GlcNAc})} \times 100 = 21\ \% \qquad \text{(E1)}$$

The degree of polymerization (DP) was calculated from the equation E2:

$$\mathrm{DP} = \frac{\mathrm{A}_{\mathrm{H}_2}(\mathrm{GlcN}) + \frac{1}{3}\mathrm{A}_{\mathrm{CH}_3}(\mathrm{GlcNAc})}{\mathrm{H}_{\mathrm{I}}(\mathrm{amf}) + \mathrm{H}_{\mathrm{I}*}(\mathrm{amf})} = 17 \qquad \text{(E2)}$$

With H_{I} and $H_{\mathrm{I}*}$ corresponding to the H_I atoms of the amf unit linked to the GlcN and GlcNAc units, respectively.

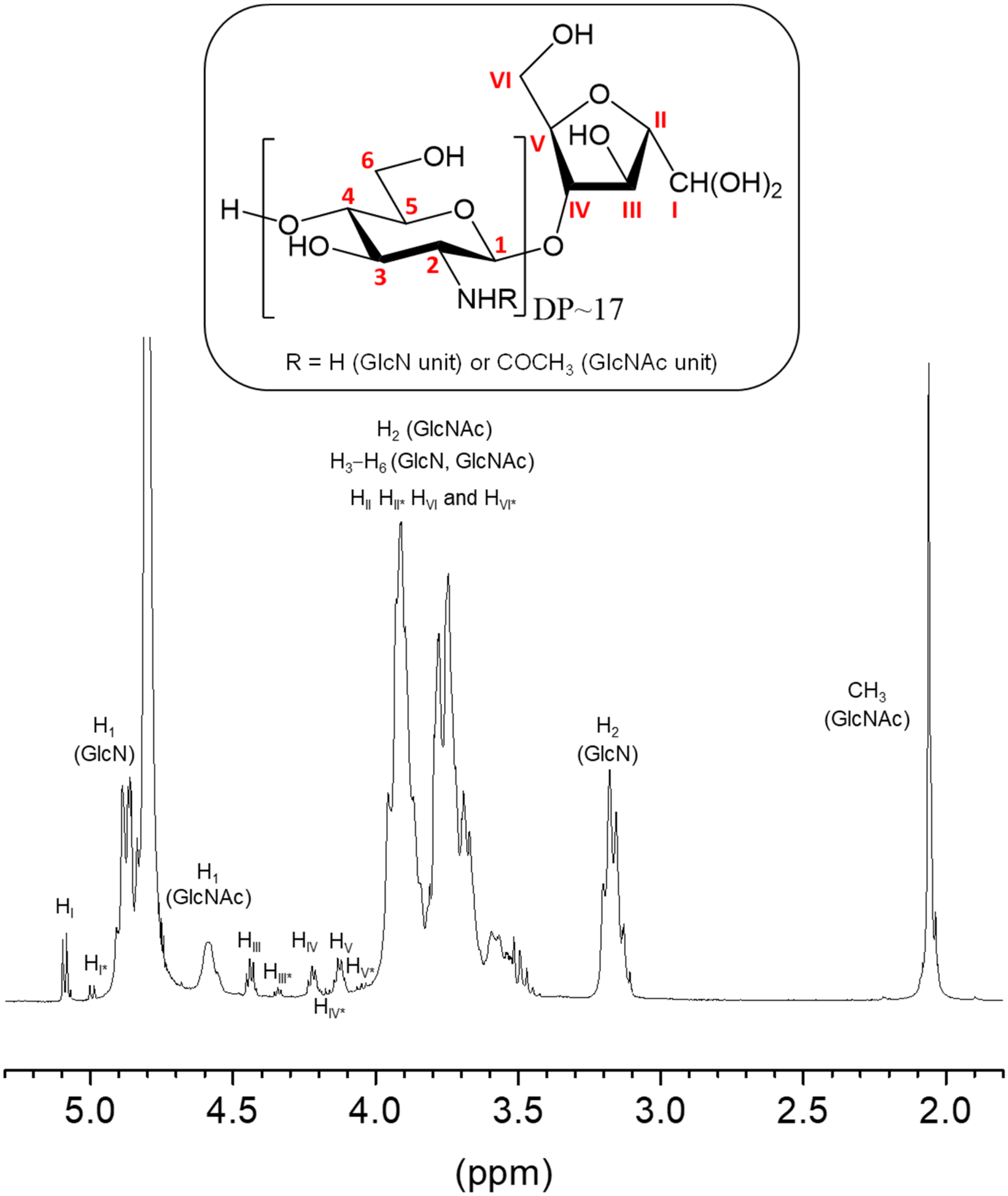

Figure S10. ^{1}H NMR spectrum of partially acetylated COSamf (DP ~17, DA ~21%) in D_2O/HCl at 300 K. Note that the gem-diol hydrated form of the amf-unit aldehyde was the only form detected [25]. H atoms of the amf unit linked to the GlcNAc unit are indicated by an asterisk.
